# Human creativity versus artificial intelligence: source attribution, observer attitudes, and eye movements while viewing visual art

**DOI:** 10.3389/fpsyg.2025.1509974

**Published:** 2025-05-12

**Authors:** Caitlin V. Cunningham, Gabriel A. Radvansky, James R. Brockmole

**Affiliations:** Department of Psychology, University of Notre Dame, Notre Dame, IN, United States

**Keywords:** art, eye movements, artificial intelligence, individual differences, attitudes, vision

## Abstract

**Introduction:**

Artificial Intelligence (AI) has the capability to create visual images with minimal human input, a technology that is being applied to many areas of daily life. However, the products of AI are consistently judged to be worse than human-created art, even when comparable in quality. The purpose of this study is to determine whether explicit cognitive bias against AI is related to implicit perceptual mechanisms active while viewing art.

**Methods:**

Participants’ eye movements were recorded while viewing religious art, a notably human domain meant to maximize potential bias against AI. Participants (*n* = 92) viewed 24 pieces of Biblically-inspired religious art, created by the AI tool DALL-E 2. Participants in the control group were told prior to viewing that the pieces were created by art students, while participants in the experimental group were told the pieces were created by AI. Participants were surveyed after viewing to ascertain their opinions on the quality and artistic merit of the pieces.

**Results:**

Participants’ gaze patterns (fixation counts, fixation durations, fixation dispersion, saccade amplitude, blink rate, saccade peak velocity, and pupil size) did not differ based on who they believed created the pieces, but their subjective opinions of the pieces were significantly more positive when they believed pieces were created by humans as opposed to AI.

**Discussion:**

This study did not obtain any evidence that a person’s explicit “valuation” of artworks modulates the pace or spatial extent of visual exploration nor the cognitive effort expended to develop an understanding of them.

## Introduction

How does knowing how something was created influence cognition and behavior? Specifically, to what degree do people treat things differently if they think they were created by a person or a machine? The main aim of the current project was to assess how the attribution of an artwork to a person or a computer-based artificial intelligence influences not only people’s opinions about it, but also how they actively perceive it. We additionally asked if any such perceptual changes vary as a function of observer attitudes toward art and artificial intelligence.

Artificial intelligence (AI) is a rapidly-growing set of technologies that enable computers to emulate many aspects of human perception, learning, comprehension, problem-solving, and decision making. Hence, they hold enormous potential to transform many aspects of daily life. However, public opinion of the growing incorporation of AI technologies in business (e.g., customer service, inventory management, supply chain operations), finance (e.g., market forecasting, fraud detection), education (e.g., adaptive learning platforms, intelligent tutoring systems), and healthcare (e.g., preventative care, medical diagnosis), is mixed. For example, a 2023 Pew Research Center survey found that 52% of Americans feel more concern than excitement about developments in AI, an increase from 37% in 2021 (Pew Research Center, 2023a). Moreover, Pew found that people want to see more regulation and oversight of AI technologies. For example, 67% do not think the government will go far enough to regulate the use of ChatBots, 75% think health care providers are moving too fast incorporating AI into their practices, and 87% want driverless vehicles to be held to higher safety testing (Pew Research Center, 2023b). Indeed, legal scholars have argued that the “race to AI” must be accompanied by a “race to AI regulation” (e.g., Smuha, 2021).

Public perception of AI also tends to be negative in areas of creative expression (e.g., Castelo et al., 2019; Köbis and Mossink, 2021; Pew Research Center, 2023c; Schepman and Rodway, 2020). The recent development of generative AI technologies has given computers the ability to create audio, text, and image content. Generative AI therefore stands to redefine creative processes that have, until now, been uniquely human. Human-created literature, film, music, dance, and art are often thought to convey the creator’s ideas, emotions, and deliberative intention to impact the viewer in some manner (e.g., Bullot and Reber, 2013; Jucker et al., 2014; Pignocchi, 2014). The arts are a way to express thoughts and communicate with others, often without words. They are valued not just for aesthetic pleasure but also the creator’s talent and originality in thought or approach. In contrast, we do not perceive generative AI as being capable of reflecting on the human condition, feeling or expressing emotions, honing talent over years of work, or developing new ways of thinking. Thus, recent cases where award-winning creative works were revealed to be AI-generated (or generated with AI assistance) have sparked extensive criticism and debate on social media platforms (see, e.g., Boris Eldagsen’s image “The Electrician” taking first place at the 2023 Sony World Photography Awards and Rie Kudan’s novel “Tokyo-to Dojo-to” winning the 2023 Akutagawa Prize).

In the visual arts, artworks attributed to an AI (or created with significant assistance from digital technologies) are generally rated lower than art attributed to a human creator in terms of aesthetics, liking, quality, novelty, meaning, and collection/purchase intention (Chamberlain et al., 2018; Chiarella et al., 2022; Gangadharbatla, 2021; Gu and Li, 2022; Hong and Curran, 2019; Kirk et al., 2009; Ragot et al., 2020; Xu and Hsu, 2020), although the magnitude of the effect can vary. For example, the bias is stronger among experts (Gu and Li, 2022), reduced when considering abstract art (Gangadharbatla, 2021), and eliminated in some viewing contexts (Chiarella et al., 2022). Regardless, within the general population, people often hesitate to call AI an “artist” or its creations “art” (Hong, 2018; Lyu et al., 2022; Mikalonytė and Kneer, 2022) and the Pew Research Center has found that while over half of Americans view AI as a major advance in fields such as medicine (59%), agriculture (54%), and meteorology (50%), fewer than a third (31%) consider it to be a major advance in art (Pew Research Center, 2023c).

While negative explicit attitudes toward AI-generated art are well documented, much less research has considered the degree to which *implicit* measures of bias (i.e., those that could detect a person’s unconscious reactions to visual input) are affected by artist attribution during their encounters with artworks, with existing work providing mixed results. For example, brain activity in the entorhinal cortex, temporal pole, and primary visual cortex is greater when observers view artworks attributed to humans compared to AI, irrespective of their explicit ratings of aesthetic value (Kirk et al., 2009). However, implicit measures of psychophysiological activation, such as electrodermal activity and heart rate, have been shown to be independent of artistic attribution (Chiarella et al., 2022). Here, we ask if people’s explicit, conscious, negative biases toward AI-generated art are mirrored by implicit, unconscious, shifts in how they *look* at that art.

When viewing artworks (or any other visual stimulus), people continuously reorient their gaze from place to place. The resulting sequences of fixations (periods of time where the eyes are relatively stationary and high-fidelity foveal vision is used to accumulate visual detail from a relatively small area of a display) and saccades (ballistic eye movements that shift the point of gaze from one place to another) are not random, but arise from the real-time information processing priorities of the visual system. These priorities, and hence the eye movement behaviors exhibited while viewing art, can change as a function of image content (e.g., DiPaola et al., 2013; Fuchs et al., 2011; Massaro et al., 2012; Quiroga and Pedreira, 2011) and style (e.g., Pihko et al., 2011; Uusitalo et al., 2009), as well as the observer’s goals (Sharvashidze and Schütz, 2020), artistic preferences (e.g., Alvarez et al., 2015; Mitschke et al., 2017; Plumhoff and Schirillo, 2009), knowledge (Bubic et al., 2017), artistic expertise (e.g., Francuz et al., 2018; Lin and Yao, 2018; Koide et al., 2015; Pihko et al., 2011; Vogt and Magnussen, 2007), and perceived challenge in understanding an artwork (e.g., Ganczarek et al., 2020).

Recently, Zhou and Kawabata (2023) provided the first evidence that viewers’ beliefs about the source of an artwork (human or AI) may be associated with their viewing behavior. In their study, participants looked at 40 artworks, half made by humans and half made by generative AI. Participants were not told how each artwork was created, but they were instead asked to categorize the works themselves based on their own intuitions about their origin. The authors found that participants’ ability to discriminate between human- and AI generated artworks was poor, but when they believed an artwork was made by a human artist they looked at it longer. This result suggested to them that explicit beliefs can also manifest as implicit shifts in viewing behavior, although they acknowledged that multiple mechanisms could be responsible for those shifts and cautioned against generalization of their findings to other image sets and viewing contexts. Our study is therefore an attempt to conceptually replicate and extensively extend Zhou and Kawabata’s investigation to test the generalizability of their conclusions and to more fully characterize the potential associations between explicit attitudes towards particular artworks and how they are implicitly viewed. As discussed in more detail below, this included the inter-relationships between explicit attitudes, temporal and spatial measures of gaze control, changes in gaze behavior over time, implicit measures of engagement and mental effort, and a variety of individual differences.

Numerous eye movement variables can be measured and related to cognitive processing of visual information. Here, we focused on gaze measures that are considered content-independent because they can be obtained without reference to the visual input itself. We concentrated on such measures for two reasons. First, they reflect general processing speed and overall implicit information gathering strategies rather than local or idiosyncratic shifts in viewing based on specific stimulus content. Second, they are more likely to generalize across stimuli, tasks, and viewing contexts than content-dependent measures (e.g., visual salience and semantic content at fixation). As such, they could potentially constitute robust indicators of implicit bias with respect to artist attribution.

The “pace” with which visual information is acquired is revealed by temporal indices of gaze control such as the number of fixations and the durations of those fixations. Longer, and thus fewer, fixations tend to be associated with more difficult or more complex visual processing as more time is needed to understand the fixated information (see Henderson, 2007 for a review). The spatial extent of visual sampling can be measured by saccade amplitude (the distance between consecutive fixations) and fixation dispersion (a measure of the spread of fixation points). Shifts in spatial aspects of gaze may reflect changes in viewing strategies. For example, observers may adopt a more “exploitative” strategy in which visual processing is concentrated on a few areas or a more “exploratory” strategy where processing is more diffusely spread across many locations (e.g., Gameiro et al., 2017; Hills et al., 2015). Additionally, gaze can be used as an implicit measure of viewer engagement with, and effort applied to, visually-based tasks. Blink rates decrease when observers are looking at content that they think is important or relevant (e.g., Ranti et al., 2020); the peak velocity of saccades is inversely related to task complexity (e.g., Di Stasi et al., 2013); and, pupil size is positively correlated with the cognitive resources needed to complete a task (e.g., Kahneman and Beatty, 1966).

In the current study, we had people view a set of 24 artworks that depicted people and events described in the Bible. To control for any actual differences that might exist between AI- and human-created art, all these artworks were created using the generative AI program DALL-E 2. Thus, none of the participants had prior experience with the pieces. In a between-subjects manipulation, half of participants were led to believe that the artworks were created by humans and the other half were told they were created by AI. Our choice to use artworks that portray religious and sacred stories stemmed from a desire to maximize the dichotomy between humans and AI. While humans are capable of experiencing spirituality, faith, and a belief in the divine, machines are not. Hence, divergence in viewers’ perceptions of the intentionality of human and AI creators (a key aspect of art appreciation, see, e.g., Bullot and Reber, 2013) should be highlighted in the context of sacred art. Additionally, religious contexts may also evoke notions of morality, which people are less likely to entrust to AI (Gogoll and Uhl, 2018; Zhang et al., 2022).

Based on prior work discussed above, we expected participants in the AI group to have more explicit negative attitudes towards, opinions of, and reactions to the artworks. Our goal was to determine if these differences in attitudes are accompanied by shifts in the pace of visual processing (fixation count and duration), the extent of spatial exploration (saccade amplitude and fixation dispersion), and the exertion of effort (blink count, peak saccade velocity, and pupil size) during viewing. If people “discount” or “devalue” AI generated artworks, and this affects viewing behavior, we may observe shifts in gaze-based hallmarks of greater visual and cognitive engagement. For example, art thought to be created by humans may enjoy fewer fixations, longer fixation durations, greater fixation dispersion, fewer blinks, higher peak velocities, and/or larger pupil size. It is, however, important to recognize that such shifts may develop over time as people view artworks. As people view art, they transition from an initial global survey of the piece aimed at understanding its compositional elements and overall gist to a more focal analysis aimed at building a complete conceptual representation of its content (Nodine and Krupinski, 2003). By considering potential changes in eye movements over time, our goal was to determine if artistic attribution has more influence on viewing behavior early (i.e., when aesthetic appreciation is prioritized) and/or late in viewing (i.e., when understanding is prioritized).

In addition to examining the potential impact of artist attribution on viewing behavior, we took the opportunity to also consider whether and how individual differences in participants’ own religiosity, desire for aesthetics, and general attitudes toward AI affect gaze behavior regardless of author attribution. It is possible that people who are more religious (i.e., interested in the content of the artworks used in this study) or are more driven by aesthetics could view the artworks differently than people who are less so. We additionally considered individual differences in people’s general attitudes towards artificial intelligence. Those who are more positive about AI may engage with AI-created artworks differently than those who are more concerned. As with artistic attribution, such individual differences may be observed by changes in spatial, temporal, and/or cognitive aspects of gaze.

## Method

### Participants

A minimum sample size was guided by Zhou and Kawabata (2023) who reported negative explicit biases toward art they believed to be created by artificial intelligence as well as correlated shifts in the distribution of fixation durations. Their study included a single sample of 34 participants. Given our 2-group between-subjects design, we set a minimum sample size of 68 participants, with a goal to collect data until the conclusion of the Spring 2024 academic term. The final sample of 92 participants was obtained, all of whom were undergraduate students at the University of Notre Dame. Demographic information is reported in the Sample Characteristics subsection of the Results. While we did not measure participants’ level of art experience or expertise, none of them were art, art history, or design majors. Hence, the variability in our sample was likely consistent with that within the non-expert population. All participants were compensated with course credit.

### Stimuli

**Figure 1:**
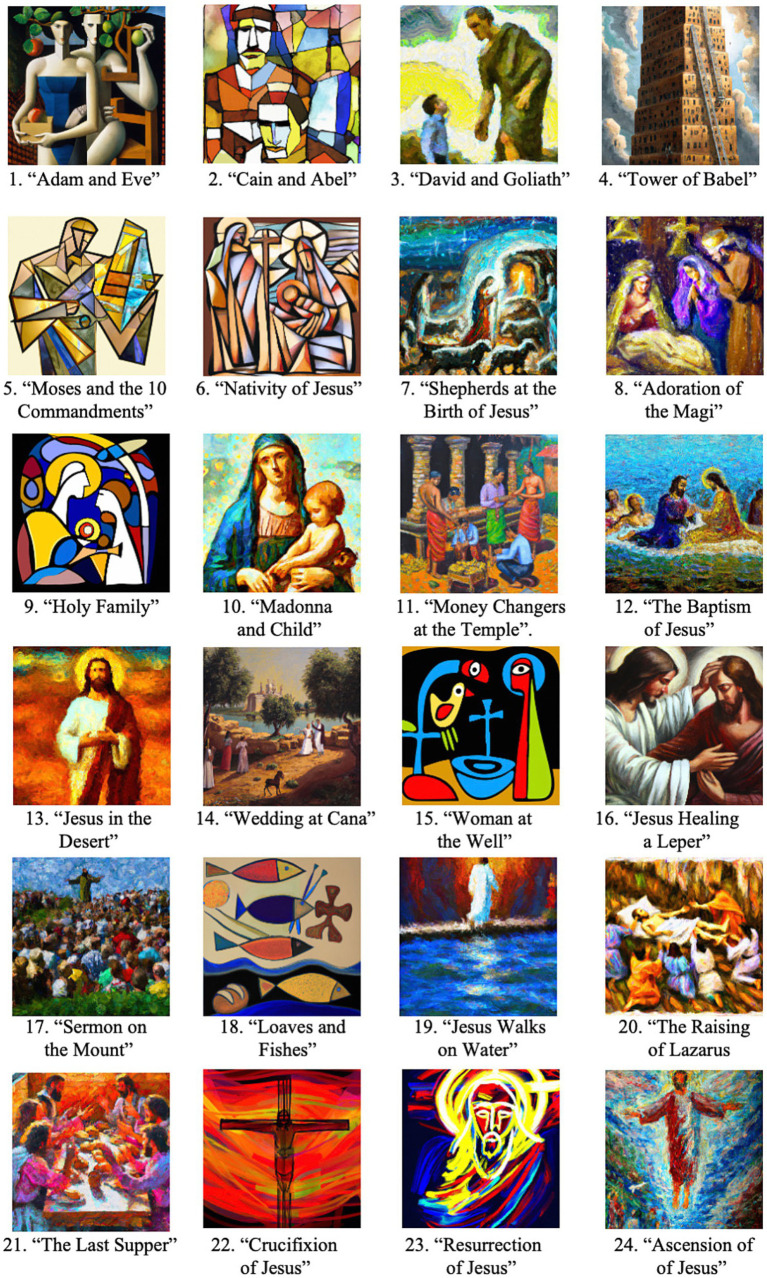
Artworks generated by DALL-E 2 and used in this study. Image numbers correspond to prompts provided in Table 1.

Stimuli consisted of 24 artworks created by DALL-E 2, OpenAI’s natural language visual art generator. Artworks were generated by providing prompts that included a topic and artistic style or artist to emulate. Examples of prompts included “painting of the Tower of Babel in a realistic style” or “painting of the loaves and fishes story in the style of Kandinsky.” Religious topics were Christian in nature, drawn from both the Old and New Testaments of the Bible. Various artistic styles were used, including Impressionism, Cubism, and realism. A full list of prompts is provided in Table 1 and the stimuli are illustrated in Figure 1.

**Table 1:** Prompts provided to DALL-E 2 to generate stimuli.

Jan Gossaert Adam and Eve painting in a cubist style. Painting of Cain and Abel in a cubist style. David and Goliath in the style of impressionism. Painting of the Tower of Babel in a realistic style. Moses holding up the Ten Commandments in the style of cubism. Jesus Nativity in the style of cubism. Jesus Nativity with shepherds in the style of impressionism. Adoration of the Magi in the style of impressionism. Holy Family in the style of Kandinsky. Raphael Madonna and Child in the style of impressionism. Oil painting of the money changers at the Temple. Painting of the Baptism of Jesus in the style of impressionism. Painting of Jesus in the desert in an impressionist style. A painting of the wedding at Cana. Jesus talking with the woman at the well in the style of Miro. Oil painting of Jesus healing a leper. Jesus on a hill talking to a crowd below in the style of impressionism. Painting of the loaves and fishes story in the style of Kandinsky. Painting of Jesus walking on water in an impressionist style. A painting of the raising of Lazarus in the style of impressionism. The Last Supper in the style of impressionism. Painting of the Crucifixion in an abstract style. Painting of the Resurrection of Jesus in an abstract style. Painting of the Ascension of Jesus in the style of Monet.

### Measures

#### Eye gaze

Eye position was recorded while participants viewed the artworks (see Apparatus). From these records, several temporal, spatial, and cognitive aspects of eye gaze were computed. Temporal parameters of gaze control included the number of fixations observers made while viewing each artwork and the duration of those fixations^1^. Spatial aspects of gaze included saccade amplitude and fixation dispersion. Saccade amplitude is the distance between consecutive fixations. Fixation dispersion is the root mean square of the Euclidean distance between each fixation point and the average position of all fixations (reported on a 0–1 scale). Gaze measures correlated with viewer engagement and cognitive effort included blink rates, the peak velocity of the eyes reached during a saccade, and pupil size.

#### Questionnaires

To assess participants’ attitudes toward the artworks, as well as the cognitive and emotional impacts of the artworks, we created a questionnaire, which, for ease of discussion, we call an *artistic impressions survey*. This survey is not presented here as a formally constructed scale, but rather as a selective amalgamation of questions drawn from (or closely inspired by) prior works that were themselves devised to understand different aspects of people’s attitudes toward artworks. Some questions were aimed at participants’ overall *appreciation* of the artworks. They were asked to indicate how much they liked the artworks (see Chamberlain et al., 2018) and how beautiful, immersive, sincere, and moving they found them to be as a whole (see Miller et al., 2024). Other questions pertained to their perception of *artistic quality* by rating originality, composition, aesthetic value, and communication of ideas (see Hong and Curran, 2019). Finally, some questions assessed the *impact* the artworks had on participants by asking them to indicate the degree to which emotions and memories were evoked (see Dageforde et al., 2024). All responses were made on a 7-point Likert-type scale. The full text of these questions and the response options are provided in Table 2. For analysis, we derived several scores based on (1) the aggregate of all responses, (2) the responses to items pertaining to art appreciation, (3) the responses to items pertaining to art quality, and (4) the responses to items pertaining to artistic impact (see Results for further details).

**Table 2:** Art impressions survey questions and response options.

Survey items
Art appreciation
1. How much did you like these pieces? 2. To what degree did you find the pieces you viewed beautiful? 3. To what degree did you find the pieces you viewed immersive? 4. To what degree did you find the pieces you viewed moving? 5. To what degree did you find the pieces you viewed sincere?
Artistic quality
6. How would you rate the pieces in their originality? 7. How would you rate the pieces in the composition (i.e., use of space)? 8. How would you rate the pieces in their aesthetic value? 9. How would you rate the pieces in their successful communication of ideas?
Artistic impact
10. To what degree were emotions evoked by the pieces your viewed? 11. To what degree were memories from your own life evoked by viewing these pieces?
Response options
Items 1–5 and 10–11: 7-point Likert-type scale with verbal labels provided for ratings of 1 (“Not at all”), 4 (“Moderately”), and 7 (“Very”). Items 6–9: 7-point Likert-type scale with verbal labels provided for ratings of 1 (“Very Poor”), 4 (“Neutral”), and 7 (“Excellent”).

Individual differences in attitudes toward aesthetics, religion, and artificial intelligence were measured with three additional questionnaires. The *Desire for Aesthetics Scale* (Lundy et al., 2010) is a 36-item questionnaire that measures individuals’ motivation to seek out and care about a wide range of aesthetic stimuli (e.g., indicating degree agreement with statements such as “I often find myself staring in awe at beautiful things.”). The *Centrality of Religiosity Scale* (Huber and Huber, 2012) measures the importance of religion and religious engagement in a person’s life (e.g., “How often do you experience situations in which you have the feeling that God or something divine intervenes in your life?”). Multiple versions of the questionnaire are available and we elected to use the 20-item interreligious version. The *General Attitudes Towards Artificial Intelligence Scale* (Schepman and Rodway, 2020) is a 20-item questionnaire that measures the degree to which a person supports, or has concerns regarding, the use of artificial intelligence in daily life. It includes both positive (e.g., indicating degree of agreement with statements such as “Artificially intelligent systems can help people feel happier.”) and negative scales (e.g., indicating degree of agreement with statements such as “Organizations use Artificial Intelligence unethically”).

### Apparatus

Artworks were presented on a 22” LCD monitor, at a resolution of 725 × 725 pixels on a dark gray 1,024 × 768 pixel background. Questionnaires were presented using Qualtrics and were presented on a Microsoft Surface Pro 8 tablet. While viewing the artworks, participants’ eye movements were sampled monocularly at a rate of 1,000 Hz using an EyeLink Portable Duo eyetracker (SR Research Inc.). Viewing distance was constrained with a chin rest positioned 95 cm from the display.

### Design and procedure

Participants were randomly assigned to one of two groups based on *artistic attribution*. The *human attribution group* (*n* = 46) was told that they would be viewing images of artworks created by students enrolled in a college course titled “Picturing the Bible” in which students study the ways Christians represented their sacred stories in visual art from the early Christian period to present day. This cover story was fictitious but believable given our extensive catalog of theology and religion-focused courses coupled with a university requirement that all undergraduate students take courses in theology and/or Catholicism. The *AI attribution group* (*n* = 46), was (truthfully) told that they would be viewing religiously themed artworks created by DALL-E 2, an artificial intelligence created by a research and development company called OpenAI that responds to natural language prompts with images.

Participants then viewed the artworks serially while their eye movements were recorded. Each trial began by presenting a “title” for each artwork (i.e., part of the prompt provided to DALL-E 2) in the center of the screen for 2 s. Then, the artwork was shown for 10 s. Between trials, participants completed a 1-point calibration to correct for drift in the eye tracker signal. The 24 items were presented in a different random order for each participant and they moved from trial to trial at their own pace. Gaze was tracked for the entire viewing phase. After viewing all artworks, participants completed the questionnaires and surveys described above, during which gaze was not tracked.

## Results

### Sample characteristics

Among participants in our sample, 62 identified as female and 30 as male. The mean age was 19.3 years (*SD* = 1.08). Sixty-eight identified as Roman Catholic, 12 as a non-Catholic Christian denomination, 2 as another Abrahamic religion (Jewish or Muslim), and 10 as having no religion.

Table 3 reports mean scores and inferential statistics obtained on the Desire for Aesthetics Scale, the Centrality of Religiosity Scale, and the General Attitudes Towards Artificial Intelligence Scale, broken down by attribution group. On the Desire for Aesthetics Scale (Lundy et al., 2010) the minimum possible score is 0 and the maximum is 216. A score of 108 indicates a neutral attitude toward aesthetics; lower scores indicate lesser, while higher scores indicate greater motivation to appreciate and incorporate aesthetics in their daily lives. On the Centrality of Religiosity Scale (Huber and Huber, 2012) the minimum possible score is 1 and the maximum is 5. Higher scores indicate religiosity is of greater, or more central, importance to a person. On the General Attitudes Towards Artificial Intelligence Scale (Schepman and Rodway, 2020) the minimum possible score is 1 and the maximum is 5. A score of 3 indicates a neutral attitude toward AI. Higher scores, regardless of subscale, indicate a more positive attitude toward AI. For none of the scales did we observe reliable differences between attribution conditions (all *p*’s > 0.48). Because these scales could not be used to differentiate participant groups, they cannot account for differences in gaze behavior based on creator attribution, should they be observed.

**Table 3:** Mean scores (with standard deviations) obtained on the desire for aesthetics, centrality of religiosity, and general attitudes toward artificial intelligence scales broken down by attribution.

	Human attribution	AI attribution	Statistics (*t*, *p*, *d*)
Desire for aesthetics	132.9 (18.8)	133.3 (20.9)	*t*(90) = 0.10, *p* = 0.92, *d* = 0.02
Centrality of religiosity	3.36 (0.79)	3.23 (0.94)	*t*(90) = 0.71, *p* = 0.48, *d* = 0.15
General Attitudes Toward AI (Positive Subscale)	3.32 (0.67)	3.33 (0.70)	*t*(90) = 0.07, *p* = 0.94, *d* = 0.02
General Attitudes Toward AI (Negative Subscale)	2.90 (0.72)	2.87 (0.67)	*t*(90) = 0.22, *p* = 0.82, *d* = 0.05

### Artistic attribution and explicit attitudes toward artworks

**Figure 2:**
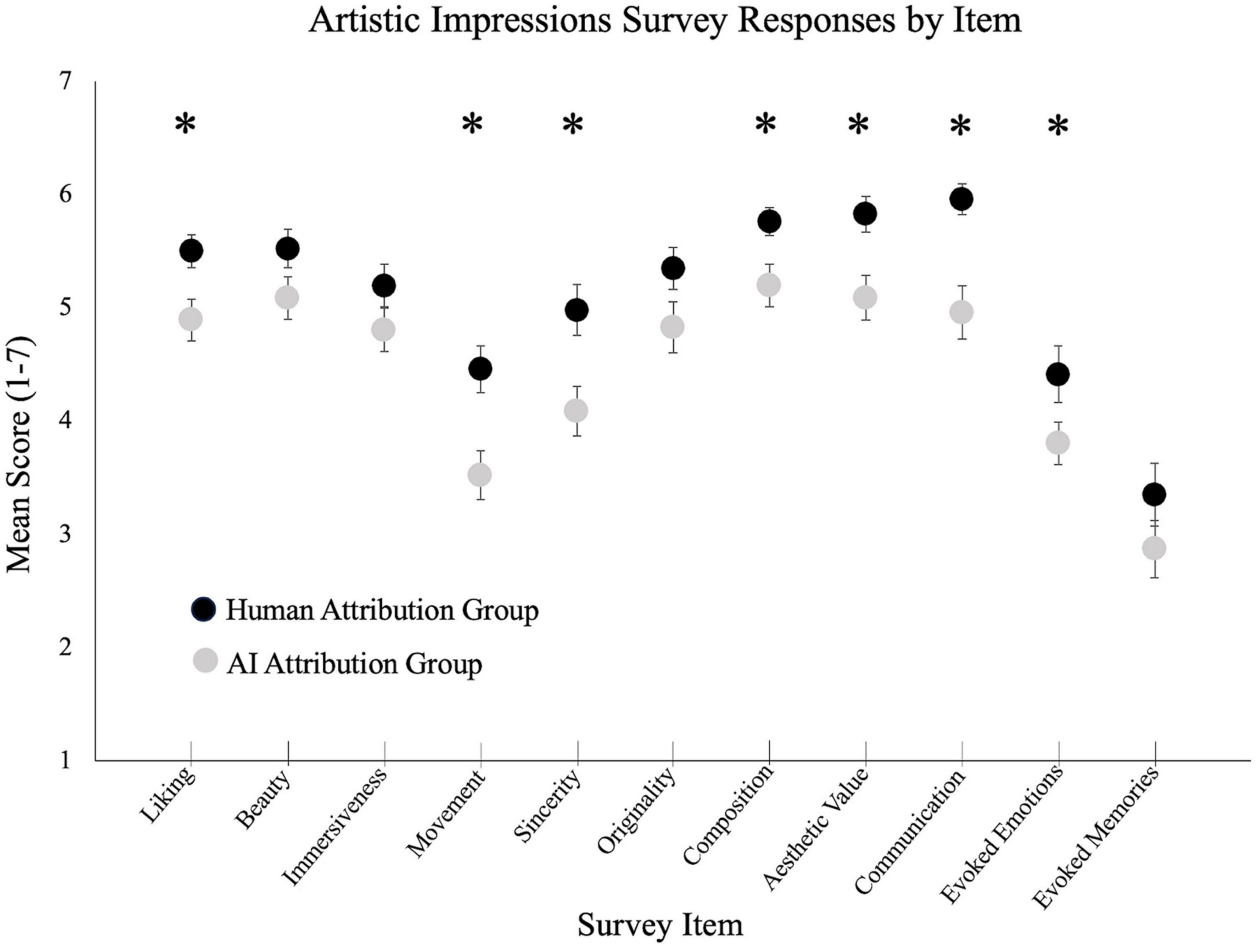
Mean responses to items included in the artistic impressions survey. Full item descriptions are provided in Table 2. Error bars depict +/− 1 standard error of the mean. Asterisks denote reliable differences between the human- and AI attribution groups (*p* < 0.05).

Responses to the individual questions included in our artistic impressions survey are summarized in Figure 2. We analyzed this data in multiple ways. First, we computed an overall *impressions score* by averaging responses across all questions. This was higher (more favorable) for the human attribution group (*M* = 5.12, *SD* = 0.80) than the AI attribution group (*M* = 4.47, *SD* = 0.97), *t*(90) = 3.52, *p* < 0.001, *d* = 0.22.

Second, we calculated an *appreciation subscore* by averaging responses to the 5 questions pertaining to participant liking, and to how beautiful, immersive, sincere, and moving they found the set of artworks to be. This subscore was higher for the human attribution group (*M* = 5.13, *SD* = 0.94) than the AI attribution group (*M* = 4.48, *SD* = 1.13), *t*(90) = 3.01, *p* = 0.003, *d* = 0.22. Within this set of questions, average scores were numerically higher on all items within the human attribution condition, with reliable differences observed for liking (*p* = 0.012), sincerity (*p* = 0.006), and movement (*p* = 0.002). Differences between groups were marginally reliable for ratings of beauty (*p* = 0.09), and not reliably different for judgments of immersiveness (*p* = 0.15).

Third, we calculated a *quality subscore* by averaging responses to the 4 questions pertaining to originality, composition, aesthetic value, and communication of ideas. This subscore was higher for the human attribution group (*M* = 5.72, *SD* = 0.67) than the AI attribution group (*M* = 5.02, *SD* = 1.09), *t*(90) = 3.75, *p* < 0.001, *d* = 0.23. Within this set of questions, average scores were numerically higher in the human attribution group for all items, with reliable differences observed for composition (*p* = 0.01), aesthetic value (*p* = 0.004), and communication of ideas (*p* < 0.001). A marginally reliable difference between groups was observed for ratings of originality (*p* = 0.08).

Fourth, we calculated an *impact subscore* by averaging responses to the 2 questions regarding evoked emotions and memories. This subscore was higher in the human attribution group (*M* = 3.88, *SD* = 1.54) than the AI attribution group (*M* = 3.34, *SD* = 1.24), but this difference was only marginally reliable, *t*(90) = 1.87, *p* = 0.07, *d* = 0.21. Within this set of questions, scores were numerically higher in the human attribution group for both items, with reliable differences observed for emotion (*p* = 0.05), but not memory (*p* = 0.21).

Finally, although we had no *a priori* hypotheses regarding the relationship between participants’ attitudes towards the artworks, their religiosity, desire for aesthetics, and attitudes towards artificial intelligence, for archival purposes Table 4 reports the correlations among these measures within each attribution group. Across groups, participants who viewed the artworks more favorably tended to more strongly incorporate aesthetics into their lives and report greater religiosity. Interestingly, in the human attribution group, participants who viewed the artworks more positively tended to have a more negative attitude toward artificial intelligence, an effect that was absent in the AI attribution group. Based on this group-level difference, we speculate that the experience of knowingly viewing AI-generated art enabled participants in the AI-attribution group to separate their evaluation of AI-generated art per se from their more general (negative) posture toward artificial intelligence as a technology.

**Table 4:** Correlations (*p* values) among survey scores.

Survey					
	Artistic impressions	Desire for aesthetics	Centrality of religion	Attitudes to AI (positive)	Attitudes to AI (negative)
Human attribution group					
Artistic imp.	-				
Desire aesth.	0.268 (0.07)	-			
Cent. relig.	0.377 (<0.01)	0.136 (0.37)	-		
AI (+)	−0.509 (<0.001)	−0.286 (0.054)	−0.381 (<0.01)	-	
AI (−)	−0.372 (0.013)	−0.262 (0.08)	−0.242 (0.11)	0.493 (<0.001)	-
AI attribution group					
Artistic imp.	-				
Desire aesth.	0.306 (0.04)	-			
Cent. relig.	0.402 (<0.01)	0.115 (0.45)	-		
AI (+)	−0.113 (0.46)	0.152 (0.31)	−0.024 (0.87)	-	
AI (−)	0.034 (0.83)	0.174 (0.25)	0.069 (0.65)	0.605 (<0.001)	-

### Artistic attribution and gaze behavior

Prior to data analysis, we established several *a priori* criteria for including gaze-based data in our analyses. Fixations that occurred outside the image borders (1.0%), fixation durations under 50 milliseconds or over 2,000 milliseconds (2.0% of fixations), saccade durations over 200 milliseconds (5.4% of saccades), and saccade amplitudes over 20 degrees (<1% of saccades) were excluded. Following these trims, 62,289 fixation samples (97.1%) and 58,828 saccade samples (94.2%) were included in the analyses.

**Figure 3:**
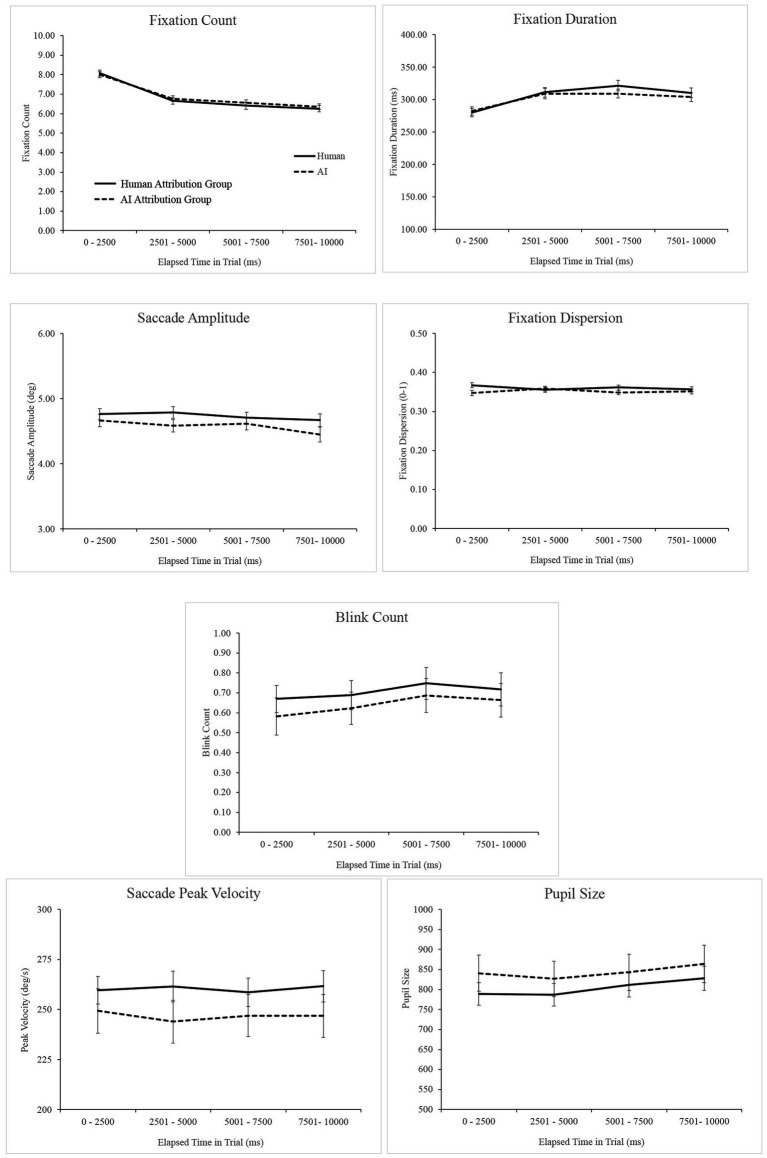
Gaze variables plotted over elapsed viewing time within trials. Error bars depict +/− 1 standard error of the mean.

Potential differences in gaze variables based on artistic attribution were assessed over time to determine if such differences emerge or diminish as viewing progresses. Each trial was divided into four 2,500 ms time windows and, within each window, average values for each gaze variable were calculated. For this within-trial analysis, gaze variables were therefore submitted to separate 2 (attribution group) × 4 (time window) mixed model analyses of variance. Data are illustrated in Figure 3 and the inferential statistics obtained from the ANOVA analyses are provided in Table 5. As viewing progressed within a trial, participants made fewer fixations, maintained fixation for longer, executed shorter saccades, and blinked more frequently. These changes over time are typical as viewing strategies shift from more global to more local visual analysis and fatigue increases. Importantly, no main effects or interactions involving art attribution were reliable^2^.

**Table 5:** Summary of main effects and interactions for ANOVA analyses of gaze measures, with art attribution as a between-subjects factor and time bin within trials as a within-subjects factor.

Gaze variable	Effect	Statistics (*F*, *p*, $\eta_{p}^{2}$)		
Temporal				
Fixation count	Attribution	*F*(1, 90) = 0.105	*p* = 0.75	$\eta_{p}^{2}$ = 0.001
	Time	*F*(3, 270) = 296	*p* < 0.001	$\eta_{p}^{2}$ = 0.767
	Attribution × Time	*F*(3, 270) = 1.28	*p* = 0.28	$\eta_{p}^{2}$ = 0.014
Fixation duration	Attribution	*F*(1, 90) = 0.242	*p* = 0.62	$\eta_{p}^{2}$ = 0.003
	Time	*F*(3, 270) = 49.9	*p* < 0.001	$\eta_{p}^{2}$ = 0.357
	Attribution × Time	*F*(3, 270) = 2.15	*p* = 0.09	$\eta_{p}^{2}$ = 0.023
Spatial				
Saccade amplitude	Attribution	*F*(1, 90) = 1.65	*p* = 0.20	$\eta_{p}^{2}$ = 0.018
	Time	*F*(3, 270) = 3.48	*p* = 0.02	$\eta_{p}^{2}$ = 0.037
	Attribution × Time	*F*(3, 270) = 0.862	*p* = 0.46	$\eta_{p}^{2}$ = 0.009
Fixation dispersion	Attribution	*F*(1, 90) = 2.88	*p* = 0.09	$\eta_{p}^{2}$ = 0.031
	Time	*F*(3, 270) = 0.146	*p* = 0.93	$\eta_{p}^{2}$ = 0.002
	Attribution × Time	*F*(3, 270) = 1.52	*p* = 0.21	$\eta_{p}^{2}$ = 0.017
Cognitive				
Peak velocity	Attribution	*F*(1, 90) = 1.16	*p* = 0.28	$\eta_{p}^{2}$ = 0.003
	Time	*F*(3, 270) = 0.248	*p* = 0.86	$\eta_{p}^{2}$ = 0.008
	Attribution × Time	*F*(3, 270) = 0.766	*p* = 0.51	$\eta_{p}^{2}$ = 0.008
Blinks	Attribution	*F*(1, 90) = 0.363	*p* = 0.55	$\eta_{p}^{2}$ = 0.004
	Time	*F*(3, 270) = 5.54	*p* < 0.001	$\eta_{p}^{2}$ = 0.058
	Attribution × Time	*F*(3, 270) = 0.166	*p* = 0.92	$\eta_{p}^{2}$ = 0.002
Pupil size	Attribution	*F*(1, 90) = 0.555	*p* = 0.46	$\eta_{p}^{2}$ = 0.006
	Time	*F*(3, 270) = 40.5	*p* < 0.001	$\eta_{p}^{2}$ = 0.310
	Attribution × Time	*F*(3, 270) = 2.61	*p* = 0.05	$\eta_{p}^{2}$ = 0.028

### Individual differences and gaze behavior

Scores on the Artistic Impressions, Desire for Aesthetics, Centrality of Religiosity, and General Attitudes Towards Artificial Intelligence Scales were correlated with each gaze variable. For this analysis we collapsed across artistic attribution, time windows within trials, and blocks of trials across the experiment. Our analysis of participants’ attitudes towards AI was restricted to the AI attribution group because this was the only group that was told the artworks were produced by a generative AI. Correlation coefficients and associated *p*-values are reported in Table 6. Within each scale, *p*-values were adjusted using the false discovery rate (Benjamini and Hochberg, 1995) to correct for multiple comparisons. None of the calculated correlation coefficients was statistically reliable. Hence, we obtained no evidence that individual differences in the importance religion and aesthetics play in people’s lives altered the way in which they viewed religiously themed artworks. Similarly, we obtained no evidence that people’s attitudes towards AI affect their viewing of AI generated art.

**Table 6:** Correlations coefficients (with FDR adjusted *p*-values) among gaze measures and survey scores.

Survey	Fixation count	Fixation duration	Fixation dispersion	Saccade amplitude	Peak velocity	Blink rate	Pupil diameter
Artistic imp	−0.104 (0.60)	0.111 (0.60)	−0.056 (0.60)	−0.058 (0.60)	0.068 (0.60)	0.123 (0.60)	−0.067 (0.60)
Desire aesth.	−0.146 (0.28)	054 (0.68)	−0.193 (0.19)	0.044 (0.68)	0.136 (0.28)	0.233 (0.19)	0.184 (0.19)
Cent. relig	−0.118 (0.52)	0.071 (0.58)	−0.133 (0.52)	−0.104 (0.52)	−0.002 (0.99)	0.094 (0.82)	−0.177 (0.52)
Attitudes toward AI (Positive subscale)	0.176 (0.61)	−0.054 (0.84)	0.322 (0.21)	0.024 (0.87)	0.141 (0.61)	−0.117 (0.61)	−0.114 (0.63)
Attitudes toward AI (Negative subscale)	−0.050 (0.82)	0.058 (0.82)	0.064 (0.82)	−0.138 (0.82)	−0.038 (0.82)	−0.062 (0.82)	0.035 (0.82)

## Discussion

Generative AI has the ability to produce content that, until recently, depended exclusively on the creative efforts of human beings. Today, algorithms can generate, in seconds (or less), original textual, audio, and visual outputs. Despite the potential utility and efficiency of such programs, people are hesitant to refer to their outputs as “art” (Hong, 2018; Lyu et al., 2022; Mikalonytė and Kneer, 2022) and consider them to have less intrinsic and extrinsic value than those created by a person (Chamberlain et al., 2018; Chiarella et al., 2022; Gangadharbatla, 2021; Gu and Li, 2022; Hong and Curran, 2019; Kirk et al., 2009; Ragot et al., 2020; Xu and Hsu, 2020). The primary goal of the study reported here was to determine if the attribution of visual artworks to AI leads people to look at them differently than when they are attributed to a human artist. Our focus was on potential shifts in the pace and extent of visual exploration, as well as the exertion of mental effort during encounters with artworks.

Participants in this study looked at a set of artworks created using DALL-E 2, a generative AI program that can produce realistic images from a natural language prompt. These artworks all depicted events described in the Old and New Testaments of the Bible. In a between-subjects manipulation, participants were either told the images were created by students in a college course called “Picturing the Bible” or truthfully told they were generated by an AI. While viewing the artworks, participants’ eye movements were recorded, and afterward, they completed surveys to ascertain their opinions of the artworks and to measure their attitudes towards religion, aesthetics, and AI.

Even though all of our participants saw *exactly* the same artworks, those in the human-attribution group had more positive opinions of them. Specifically, participants in this group judged the artworks to be more likable, sincere, and moving; they found them to be better composed, to have higher aesthetic value, and to more effectively communicate ideas to the viewer; and, they felt that the artworks evoked stronger emotional responses. These findings are consistent with prior work (Chamberlain et al., 2018; Chiarella et al., 2022; Gangadharbatla, 2021; Gu and Li, 2022; Hong and Curran, 2019; Kirk et al., 2009; Xu and Hsu, 2020), and indicate that people’s appreciation and evaluation of visual artworks, as well as their affective response to them, are affected by the knowledge they have about the human- or machine-made origin of those works. Such results are also consonant with studies that have shown that viewers’ impressions of artworks can be modulated by other contextualizing information provided alongside artworks such as titles (Millis, 2001), explanatory labels (Temme, 1992), and artist statements (Specht, 2010), as well as prior knowledge of the artist including his or her prestige (Mastandrea and Crano, 2019), personality (Van Tilburg and Igou, 2014), nationality (Mastandrea et al., 2021), disability (Szubielska et al., 2020), and other biographical details (e.g., Kaube and Rahman, 2024).

The differences we observed in participants’ attitudes toward artworks attributed to humans and AI were not accompanied by any changes in how they viewed the artworks. Fixation counts, fixation durations, fixation dispersion, saccade amplitude, blink rate, saccade peak velocity, and pupil size were unaffected by art attribution. Hence, we did not obtain any evidence to suggest that a person’s explicit “valuation” of artworks modulates the pace or spatial extent of visual exploration nor the cognitive effort needed or expended to develop an understanding of them. We were also unable to detect group differences in viewing behavior over time which further suggests that early exploratory viewing associated with aesthetic appreciation and later analytical viewing associated with understanding (cf. Nodine and Krupinski, 2003) are both insulated from conscious biases based on artistic attribution.

Our results provide some replication of, and contrast to, prior research seeking to connect artistic attribution to viewing behavior. Recently, Zhou and Kawabata (2023) considered fixation counts, individual fixation durations, and the cumulative time spent looking at images of artworks, some of which were human-created and others of which were AI-generated. When participants judged a work to be human-generated, their cumulative looking (dwell) times were, on average, 330 ms longer per image (the available viewing window was 20 s). That said, as in our study, fixation counts and individual fixation durations were not affected. For dwell times to increase without a corresponding increase in fixation counts and/or individual fixation durations is curious. One possibility is that the effect of artistic attribution may be too small to be reliably detected at the level of the individual fixations, and, instead, may only be observed when fixations are aggregated. To test this hypothesis in our own data, we calculated the total duration of all fixations made by our participants. Summing across all stimuli and fixations, participants in the human attribution group looked at the artworks for an average of 195,654 ms, or about 3 min and 15 s (SD = 14,672 ms), and those in the AI attribution group looked at them for 196,620 ms (SD = 14,805 ms) (*M_diff_* = 34 ms, *p* > 0.99). Hence, when aggregating fixations in our study we failed to replicate Zhou and Kawabata’s (2023) dwell time finding and have found no empirical support for their conclusion that gaze behavior can be affected by artistic attribution to humans or computers.

In addition to the effects of artistic attribution on how people look at art, we also considered, as a secondary question, the degree to which some individual differences might also affect viewing behavior. To this end, we measured participants’ own religiosity, desire for aesthetics, and general attitudes toward AI and correlated these with gaze behaviors. None of these individual differences correlated with any measure of viewing behavior. Hence, we obtained no evidence to suggest that individual differences in participant attitudes toward the subject matter of artworks, aesthetics, or artificial intelligence altered the way in which they viewed artworks.

**Figure 4:**
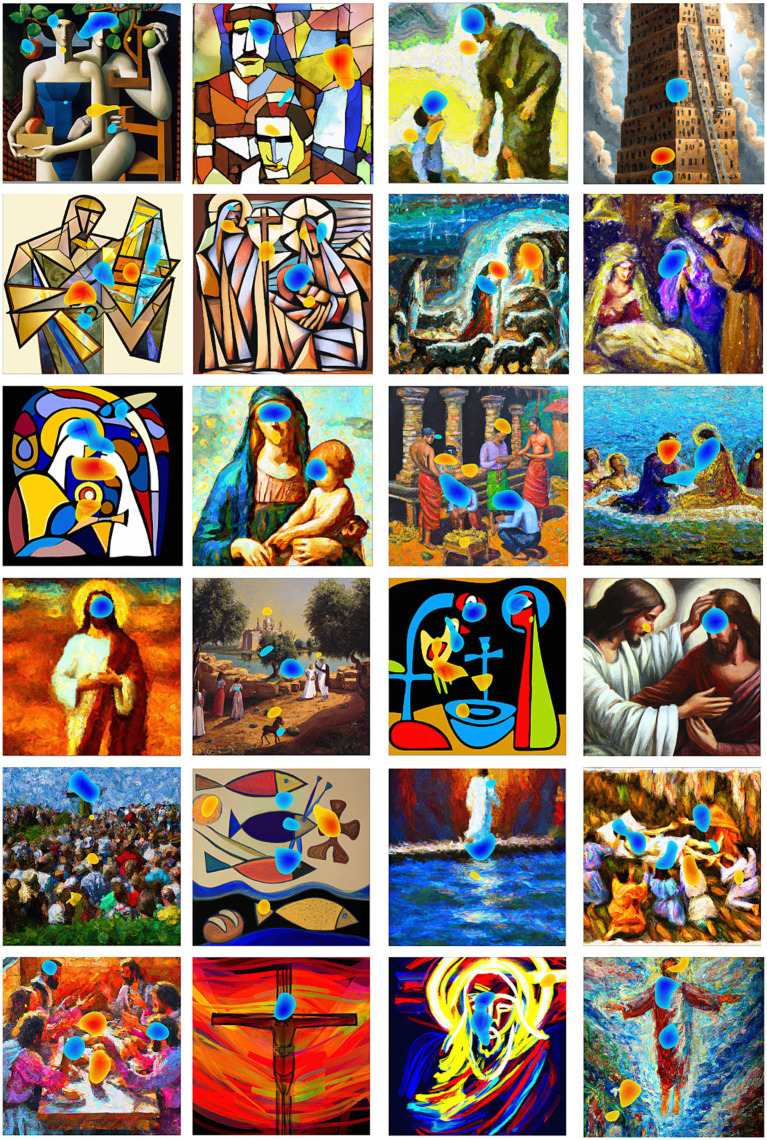
Yellow and red tones denote areas looked at longer in the AI attribution condition whereas blue tones mark areas that were looked at longer in the human attribution condition (see Footnote 3 for additional detail).

While we observed no effects of artistic attribution and various individual differences on gaze, it is possible that other empirical approaches could demonstrate such links. First, our choice to focus on content-independent gaze parameters was motivated by our goal to consider implicit, generalizable, viewing strategies divorced from idiosyncratic differences in stimulus content. Future work could consider content-dependent measures related to the visual and semantic content of artworks. If one’s beliefs about the origins of art modify the balance between visual or semantic analysis, it may be that the degree to which gaze is driven by factors such as visual salience, edge density, and semantic informativeness could be altered. Indeed, an unplanned exploratory analysis of the current data suggests that such differences in content-dependent measures may exist^3^. Figure 4 depicts those areas within each image that were disproportionately fixated in the AI attribution conditions relative to the human attribution condition and vice versa. Areas marked in yellow and red tones were looked at longer in the AI attribution condition whereas areas marked in blue tones were looked at longer in the human attribution condition^4^. One striking commonality across the artworks was a propensity for participants to spend more time looking at faces when they thought the artworks were created by humans. Second, we chose to constrain viewing at a constant distance, for a set period of time, over a predetermined group of images. In many cases, however, when viewing art these parameters are not so limited. Future work could investigate the possibility that artist attribution and/or viewers’ interest in the content of art, their appreciation of aesthetics, and/or their attitudes towards AI could lead them to make different overt choices regarding how closely they might approach an artwork, how long they might look at them, or even which artworks to look at or pass by altogether. Third, while our participant sample was representative of the general public, we were unable to determine how the relationship between one’s attitudes toward art and gaze behavior might vary as a function of artistic expertise, a factor known to influence viewing (e.g., Francuz et al., 2018; Lin and Yao, 2018; Koide et al., 2015; Pihko et al., 2011; Vogt and Magnussen, 2007). Finally, aside from our exploratory artwork-level analysis described above, our measures of both explicit attitudes and implicit viewing behaviors collapsed across individual artworks, restricting our conclusions to those that can be drawn about the collection of works as a whole. In future work, a set of images could be constructed to specifically explore the possibility that more fine-grained differences will emerge between individual items that vary in artistic style, subject matter, image content, or other factors that differentiate one work from another in either its physical composition or its influence on individual participants (e.g., observer goals, artistic preferences, knowledge, etc.).

In summary, while recent studies (including this one) have consistently found explicit negative biases against AI-generated art, we found no evidence that such biases are mirrored by implicit shifts in how non-expert viewers look at such art. Moreover, we found no evidence that individual differences in attitudes toward content, aesthetics, and AI alter looking behavior. Together, these results suggest that factors known to influence the specific content that we choose to look at (e.g., knowledge, personal attitudes and opinions) do not affect content-independent aspects of gaze related to the pace, spatial extent, and effort of visual exploration.

## Data Availability

The raw data supporting the conclusions of this article will be made available by the authors, without undue reservation.

## Footnotes

^1^Measures of frequency and duration are not orthogonal. Within a given amount of time, an increase in fixation durations should correlate with a decrease in the number of fixations observed. The measures do, however, characterize gaze in different ways and consistency across non-orthogonal measures enables stronger conclusions to be drawn from the data.

^2^We additionally conducted an items-level analysis and no difference in any measure of gaze was observed between the human- and AI-attribution conditions for any item, indicating consistency in our findings across our materials.

^3^We thank an anonymous reviewer for suggesting this exploratory analysis.

^4^Each image in Figure 4 was generated using the “difference fixation map” function in EyeLink Data Viewer Version 4.1.63. These fixation maps collapse across participants and are based on the cumulative duration of all fixations. In order to highlight areas of greatest difference across the attribution conditions, the low activity cut-off percentage was set to 40%. All other parameters were set to default values. Note that like-colors indicate equivalent differences within, but not across, images.
